# Department of Defense Response to a Multistate Outbreak of Fungal Meningitis — United States, October 2012

**Published:** 2013-09-27

**Authors:** Amy Peterson, Leslie Clark, Gi-Taik Oh, Julie Pavlin, Kevin L. Russell, Rohit A. Chitale

**Affiliations:** Armed Forces Health Surveillance Center, Silver Spring, MD

On October 1, 2012, the Department of Defense (DoD) learned of a multistate outbreak of fungal meningitis in persons who received injections of methylprednisolone acetate (MPA) from a single compounding pharmacy. Ten patients with fungal meningitis after epidural steroid injection (ESI) were initially identified in Tennessee and North Carolina (1,2). No military treatment facilities had received MPA from this pharmacy. However, clinics receiving implicated MPA lots were located throughout the United States, and active duty military service members and other DoD health-care beneficiaries could have been exposed through health-care services purchased outside of the DoD health-care system. Therefore, a timely method was needed to determine whether exposure to implicated MPA had occurred among DoD personnel who used purchased care.*

Although the majority of medical record data from outpatient treatment at military facilities are available in the Defense Medical Surveillance System (DMSS) within 7–10 days, data from procedures obtained through purchased care typically are not available for 4–6 months, and sometimes for as much as 1 year. Patient notification and reporting of cases to CDC is handled through state health departments. However, cases among highly mobile military members (who are commonly deployed and relocated) might not have been detected through standard local and state public health channels. Additionally, fungal infections can have a long incubation period (3,4) requiring a prolonged investigation to determine whether infection occurred. In response, the Armed Forces Health Surveillance Center (AFHSC), working with Tricare Management Activity, which manages medical and dental programs for DoD health-care beneficiaries, initiated an investigation to 1) identify service members and other beneficiaries who had received an injection of MPA from clinics named in the investigation, 2) determine whether any recently deployed service members had been exposed to implicated MPA, and 3) identify cases of infection among those who had been exposed.

Exposure was defined as a steroid injection into sterile epidural or joint space at clinics that received implicated MPA during the CDC-defined risk period (5). A case was defined as development of fungal infection in a joint injection site in persons meeting the exposure criteria. Tricare regional offices compiled a line-list of exposed military members by combining submitted claims data with a request for unsubmitted claims data for the defined procedures from clinics that received lots of the implicated MPA. AFHSC developed an ongoing, prospective search within DMSS to track exposed beneficiaries for possible fungal infection outcomes (6).

The results of the investigation determined that 471 military members and other beneficiaries had received potentially contaminated epidural or sterile joint injections; 43.9% were male. Of 469 persons with military status reported, 64 (13.6%) were active duty, and 58 (90%) of those were men. Among active duty service members receiving an injection with MPA, three (5%) deployed within a period in which they were at risk for a fungal infection; one deployed service member developed a fungal infection after the injection and was medically evacuated. Overall, four cases were detected in military members; three of these persons developed meningitis, including two who were active duty service members. As of November 2, 2012, no new cases had been detected through ongoing surveillance.

This investigation used a unique approach to identify cases of fungal meningitis, combining 1) claims data used to identify exposed persons and track them during relocations that included deployments and 2) ongoing outcome surveillance for additional cases within DMSS. Within DoD, universal access to care and centralized electronic medical records along with a unified system for purchased care allowed for a different approach to health surveillance in the military population, which consists of service members (active duty, Reserve, and National Guard), their dependents, and retirees. Although this approach might not apply to the entire U.S. population, it could be used by practitioners performing disease investigations and surveillance within specific groups such as managed-care programs and other large, linked databases, public and private.

## Footnotes

* “Purchased care” refers to health care received by DoD military members and other beneficiaries from civilian providers outside of military treatment facilities. Health care received at military facilities is referred to as “direct care.”
